# Attenuation of CCl_4_-Induced Hepatic Fibrosis in Mice by Vaccinating against TGF-β1

**DOI:** 10.1371/journal.pone.0082190

**Published:** 2013-12-11

**Authors:** Xiaobao Fan, Qiannan Zhang, Shuang Li, Yifei Lv, Houqiang Su, Huiping Jiang, Zhiming Hao

**Affiliations:** Department of Gastroenterology, the First Affiliated Hospital, School of Medicine, Xi’an Jiaotong University, Xi’an, China; National Institutes of Health, United States of America

## Abstract

Transforming growth factor β1 (TGF-β1) is the pivotal pro-fibrogenic cytokine in hepatic fibrosis. Reducing the over-produced expression of TGF-β1 or blocking its signaling pathways is considered to be a promising therapeutic strategy for hepatic fibrosis. In this study, we evaluated the feasibility of attenuating hepatic fibrosis by vaccination against TGF-β1 with TGF-β1 kinoids. Two TGF-β1 kinoid vaccines were prepared by cross-linking TGF-β1-derived polypeptides (TGF-β1^25^–[41-65] and TGF-β1^30^–[83-112]) to keyhole limpet hemocyanin (KLH). Immunization with the two TGF-β1 kinoids efficiently elicited the production of high-levels of TGF-β1-specific antibodies against in BALB/c mice as tested by enzyme-linked immunosorbent assay (ELISA) and Western blotting. The antisera neutralized TGF-β1-induced growth-inhibition on mink lung epithelial cells (Mv1Lu) and attenuated TGF-β1-induced Smad2/3 phosphorylation, α-SMA, collagen type 1 alpha 2 (COL1A2), plasminogen activator inhibitor-1 (PAI-1) and tissue inhibitor of metalloproteinase-1 (TIMP-1) expression in the rat hepatic stellate cell (HSC) line, HSC-T6. Vaccination against TGF-β1 with the kinoids significantly suppressed CCl_4_-induced collagen deposition and the expression of α-SMA and desmin, attenuated hepatocyte apoptosis and accelerated hepatocyte proliferation in BALB/c mice. These results demonstrated that immunization with the TGF-β1 kinoids efficiently attenuated CCl_4_-induced hepatic fibrosis and liver injury. Our study suggests that vaccination against TGF-β1 might be developed into a feasible therapeutic approach for the treatment of chronic fibrotic liver diseases.

## Introduction

Hepatic fibrosis is a common and central pathological process in chronic diffuse liver diseases. Excessive production and reduced degradation of the extracellular matrix (ECM), including the fibrillar type I and III collagens, proteoglycans and glycoproteins, result in the accumulation of hepatic ECM, which further disrupts the hepatic architecture by forming dense fibrous scars that encase nodules of regenerating hepatocytes, and eventually leads to cirrhosis. Elimination of the injurious stimulus is the obvious first choice for interrupting liver fibrosis. However, in most cases, removing the cause of liver fibrosis is quite difficult or even impossible. Moreover, progression of fibrosis can still persist even after the cause is eliminated. Hence, specific anti-fibrotic therapy is essential for managing chronic liver diseases. Unfortunately, few effective, safe and convenient approaches are clinically available [1,2].

Activation of hepatic stellate cells (HSCs) is the central event in hepatic fibrosis. Transforming growth factor β1 (TGF-β1) is confirmed to be the most potent stimulus for the activation of HSCs [1,3]. In addition to promoting the activation of HSCs, TGF-β1 has been demonstrated to promote apoptosis and suppress the regeneration of hepatocytes [4,5]. Therefore, inhibiting the pro-fibrotic effect of TGF-β1 is considered a promising therapeutic strategy for hepatic fibrosis. A number of studies have attempted to inhibit hepatic fibrosis by abrogating the pro-fibrotic effect of TGF-β1. These studies have used different approaches, including reducing the synthesis of active TGF-β1 by gene silencing [6] or through the expression of protease inhibitors [7], neutralizing TGF-β1 through treatment with specific antibodies (Ab) [8,9], creating TGF-β1 sinks with soluble TGF-β receptors [10-12] or truncated TGF-β receptors [13,14], blocking ligand-receptor interaction by TGF-β1-specific polypeptide [15], and suppressing the post-receptor signal transduction pathways [16]. Although the efficacies of these measures have been validated in experimental hepatic fibrosis, their feasibility in clinical therapeutic practice is questionable. Some of the agents mentioned above have short half-lives that require repeated administration over a long time period to achieve therapeutic benefits. Methods involving genetic modification are associated with safety concerns. Considering that clinical hepatic fibrosis is a persistent, chronic process, only a safe, effective and convenient measure for the continuous elimination of TGF-β1 is feasible for treating hepatic fibrosis. 

Vaccines against pathological cytokines or growth factors are appreciated as a “new generation of therapeutic vaccines” [17,18] and have been investigated in a number of disease models and clinical trials [19-30]. By cross-linking or creating fusion proteins with carrier proteins, the normally non-antigenic cytokines or growth factors can be converted into self-antigens to elicit specific Abs [31] to neutralize abnormally overproduced cytokines or growth factors and to inhibit their deleterious effects in pathological tissues. Here, we report that immunization with two TGF-β1 kinoids, which are prepared by cross-linking two fragments of TGF-β1-derived polypeptide with keyhole limpet hemocyanin (KLH), elicits the production of a high titer of neutralizing autoantibodies against TGF-β1 and significantly suppresses CCl_4_-induced hepatic fibrosis in BALB/c mice.

## Materials and Methods

### Polypeptide design, synthesis and preparation of kinoids

Two polypeptide fragments, TGF-β1-^41^ANFCLGPCPYIWSLDTQYSKVLALY^65^ (TGF-β1^25^-[41-65]) and TGF-β1-^83^LEPLPIVYYVGRKPKVEQLSNMIVRSCKCS^112^ (TGF-β1^30^-[83-112]), were selected from the mature human TGF-β1 amino acid sequence. These polypeptide fragments are highly homologous to the corresponding fragments of mouse TGF-β1 and have been reported to be the key sequences mediating the binding interactions between TGF-β1 and its receptors [32,33]. The TGF-β1 kinoids were prepared by cross-linking these polypeptides to KLH with a 1-ethyl-3-[3-dimethylaminopropyl]carbodiimide hydrochloride (EDC) cross-linking kit (Imject® Immunogen EDC Kit with KLH, Pierce, Rockford, IL) according to the manufacturer’s protocol. The conjugates were finally desalted using D-Salt™ dextran desalting columns. Aliquots of the conjugates were stored at –20°C until use.

### Animals and experimental protocol

Specific pathogen-free, 6-week-old male BALB/c mice were provided by the Experimental Animal Center, School of Medicine, Xi’an Jiaotong University. All animals received humane care, and the experimental protocol was approved by the Institutional Animal Ethics Committee of Xi'an Jiaotong University (Permit Number: 2011-54).

To test the immunogenicity of the TGF-β1 kinoids, 24 mice were assigned to four equal groups: TGF-β1^25^ kinoid, TGF-β1^30^ kinoid, KLH, and phosphate-buffered saline (PBS). Mice in the two kinoid groups were given 4 intraperitoneal (i.p.) injections, two weeks apart, of the kinoids (50 μg in 0.2 mL). Complete Freund's Adjuvant (Sigma-Aldrich, St. Louis, MO) was used for the first immunization, and Incomplete Freund's Adjuvant (Sigma) was used for all subsequent immunizations. The control groups were treated similarly, except that the kinoid was replaced by KLH (50 μg in 0.2 mL) or PBS. Blood samples were collected from the tail vein for ELISA immediately preceding each immunization. Two weeks after the fourth injection, three mice in each group were euthanized and the blood was collected to isolate the serum for Western blotting and the neutralization assay. The remaining mice were maintained, and blood samples were taken every two weeks for ELISA detection of the Ab titer. Six months after the first immunization, the mice were euthanized. The liver, lungs, heart and kidneys were harvested to evaluate any adverse effects.

In the fibrosis experiment, 54 male BALB/c mice were assigned to five groups: TGF-β1^25^ kinoid/CCl_4_ (n = 10), TGF-β1^30^ kinoid /CCl_4_ (n = 10), KLH/CCl_4_ (n =8), CCl_4_ (n=8) and normal control (NC) (n=8). The mice were maintained and immunized as described in the above experiment except that the latter two groups were given PBS instead of immunogens. One week after the third immunization, the mice in the former four groups received i.p. injections of CCl_4_ (1 mL/kg, dissolved in olive oil to reach a final concentration of 20%) twice a week for 6 weeks. The mice in the NC group were dosed with an equal volume of olive oil. After 6 weeks of CCl_4_ injections, the mice were euthanized. The left lobe of the liver was fixed with 4% paraformaldehyde for histological examination. The right lobe was snap-frozen in liquid nitrogen and stored at –70°C for hydroxyproline content determination and phosphorylated Smad2/3 detection. The serum was collected for ELISA determination of the titers of anti- TGF-β1 Abs and for the evaluation of their inhibitory effects on the TGF-β1 signaling, transdifferentiation and ECM secretion in the HSCs.

### ELISA determination of serum anti–TGF-β1 Ab

Polystyrene microplates were coated with commercially available, biologically active human TGF-β1 (R&D Systems, Minneapolis, MN), TGF-β2 (ProSpec-Tany TechnoGene Ltd, Ness Ziona, Israel) or TGF-β3 (ProSpec-Tany TechnoGene Ltd) at 20 ng/well. Upon detection, the mouse serum was serially diluted (1:2, initiated at 1:20) and added to the wells. Horseradish peroxidase (HRP)-conjugated goat anti-mouse IgG (Sigma) was used as the secondary Ab. A reaction was considered positive if optical density (OD) ≥ 2.1 times of the negative control.

### Western blotting

To test the specificity of the antibodies elicited by vaccination with the kinoids, 5 μg of prokaryotically expressed human TGF-β1 (prepared by our group) was applied to a 12% SDS–PAGE and transferred onto a nitrocellulose membrane. The membrane was incubated with mouse serum that was diluted 1:800 at 4°C overnight, followed by rinsing three times with TBS containing 0.1% Tween–20 (TBST). Then, the membrane was incubated with HRP-conjugated goat anti-mouse IgG (Sigma) for 1 hour. The blots were detected using an enhanced chemiluminescence solution.

 To detect α-SMA, MMP-1 and phosphorylated Smad2 and Smad3 (pSmad2/3) in the mouse liver tissues and cultured HSC-T6, the cells and liver tissues were lysed in radioimmunoprecipitation (RIPA) lysis buffer supplemented with phosphatase and protease inhibitors. A total of 100 μg of the protein was applied to SDS-PAGE. The primary antibodies used were mouse anti–α-SMA mAb (Lab Vision, Fremont, CA), rabbit anti-matrix metalloproteinase-2 (MMP-2) pAb (Abcam Inc. Cambridge, MA) and goat anti-pSmad2/3 (Ser423/425) antibody (Santa Cruz Biotech Inc., CA, sc-11769). The expression of β-actin (mouse anti–β-actin mAb, Sigma) and Smad2/3 (rabbit anti-Smad2/3 antibody, Cell Signaling Technology, Danvers, MA) served as the internal controls.

### Neutralization test

Growth assays with the mink lung epithelial cells (Mv1Lu, ATCC CCL-64) were performed by measuring bromodeoxyuridine (BrdU) incorporation (BrdU Cell Proliferation Assay; Calbiochem, San Diego, CA). Mv1Lu cells were plated on 96-well plates at 3 × 10^3^/well in complete Dulbecco’s modified Eagle’s medium (DMEM, Gibco BRL, Carlsbad, CA, USA) for 12 hours. The cells were then starved in serum-free medium for 48 hours. The starvation medium was removed and replaced with fresh medium containing various dilutions of the antiserum, 2.5 ng of TGF-β1 and BrdU. Forty-eight hours post-treatment, BrdU incorporation in the cells was detected by an anti-BrdU Ab and quantified by ELISA according to the manufacturer’s instructions.

 To test the effects of the antisera on the HSCs, a rat HSC cell line, HSC-6T, was inoculated in six-well plates at 1 × 10^6^/well. After 24 hours, the medium was replaced with DMEM supplemented with 5% FBS. TGF-β1 (6 ng/ml) and the mouse serum (1:200) were added and the cells were maintained for 24 hours. Then, the cells were harvested for the detection of α-SMA, collagen type I alpha 2 (COL1A2), plasminogen activator inhibitor-1 (PAI-1) and tissue inhibitor of metalloproteinase-1 (TIMP-1).

### Real-time quantitative reverse transcription PCR (RT-qPCR)

Total RNA was extracted using Qiagen RNeasy mini kit. Reverse transcription was performed with PrimeScript II 1st strand cDNA synthesis kit (Takara, Dalian, China). The primers used were listed in Table 1. Real-time PCR reactions were carried out using iQ^TM^ multicolor real-time PCR detection system (Bio-Rad, USA). Cycle threshold values were obtained from the Bio-Rad iQ5 2.0 standard edition optical system software (Bio-Rad, USA). Data were analyzed using the ΔΔCT method and β-actin served as an internal control. For each cDNA specimen, three paralelle PCR reactions were conducted in triplicate. The results were presented as mean ± SEM of three separate experiments.

**Table 1:** Primers for RT-qPCR.

Rat COL1A2	Forward	5’-AAGGGTCCTTCTGGAGAACC-3’
	Reverse	5’-TCGAGAGCCAGGGAGACCCA-3’
Rat PAI-1	Forward	5’-CAGCGCCTGTTCCACAAGTC-3’
	Reverse	5’-TGTCGTACTCGTGCCCATCC-3’
Rat TIMP-1	Forward	5’-CATCTCTGGCCTCTGGCATC-3’
	Reverse	5’-CATAACGCTGGTATAAGGTGGTCTC-3’
Rat β-actin	Forward	5’-GGAGATTACTGCCCTGGCTCCTA-3’
	Reverse	5’-GACTCATCGTACTCCTGCTTGCTG-3’

### Histological evaluation

Five-micron-thick liver sections were processed by both H&E staining and Masson’s trichrome staining to assess the architectural alterations and hepatic collagen deposition (fibrosis). The degree of fibrosis was evaluated semi-quantitatively using the Ishak system [34].

### Immunohistochemistry

Immunohistochemistry was performed using the Histostain^TM^-Plus SP kit with mouse anti–α-SMA mAb, rabbit anti-desmin polyclonal Ab, or mouse anti-PCNA mAb (all from Lab Vision, Fremont, CA) as the primary antibodies. The negative controls were performed by replacing the primary antibodies with pre-immune mouse or rabbit serum.

Computer-assisted semi-quantitative analysis was used to evaluate the areas of both positive α-SMA and desmin staining using Image-ProPlus version 4.5 (Media Cybernetics, Silver Spring, MD). The data for the α-SMA and desmin staining were expressed as the mean percentage of the positively stained area over the total tissue section area.

 PCNA expression was represented as the PCNA labeling index (PCNA LI) which was determined by blindly counting the positively stained hepatocytes out of 1000 cells in 10 randomly selected fields centered on a centrilobular vein at 400 × magnification.

### Hepatic hydroxyproline content

The total hydroxyproline content in the liver was determined as described previously [35]. The hydroxyproline content was expressed as μg/mg wet liver weight.

### Terminal deoxynucleotidyl transferase-mediated dUTP-biotin nick end labeling assay (TUNEL)

Apoptotic hepatocytes were labeled *in situ* using a TUNEL peroxidase detection kit (DeadEnd™ Colorimetric TUNEL System, Promega) according to the manufacturer’s protocol. The apoptosis index (AI) was expressed as the percentage of TUNEL-positive hepatocytes using the same method for evaluating PCNA LI.

### Statistical analysis

The quantitative data are expressed as the mean ± standard error of the mean (SEM). To assess the statistical significance of the inter-group differences in the quantitative data, Bonferroni’s multiple comparison tests were performed after one-way analysis of variance (ANOVA), followed by Bartlett’s tests to determine the homology of variance. The nonparametric data were analyzed by the Mann-Whitney *U*-test. *P* < 0.05 was considered to be statistically significant.

## Results

### Immunization with TGF-β1 kinoids efficiently elicits specific TGF-β1 neutralizing Abs in mice

**Figure 1:**
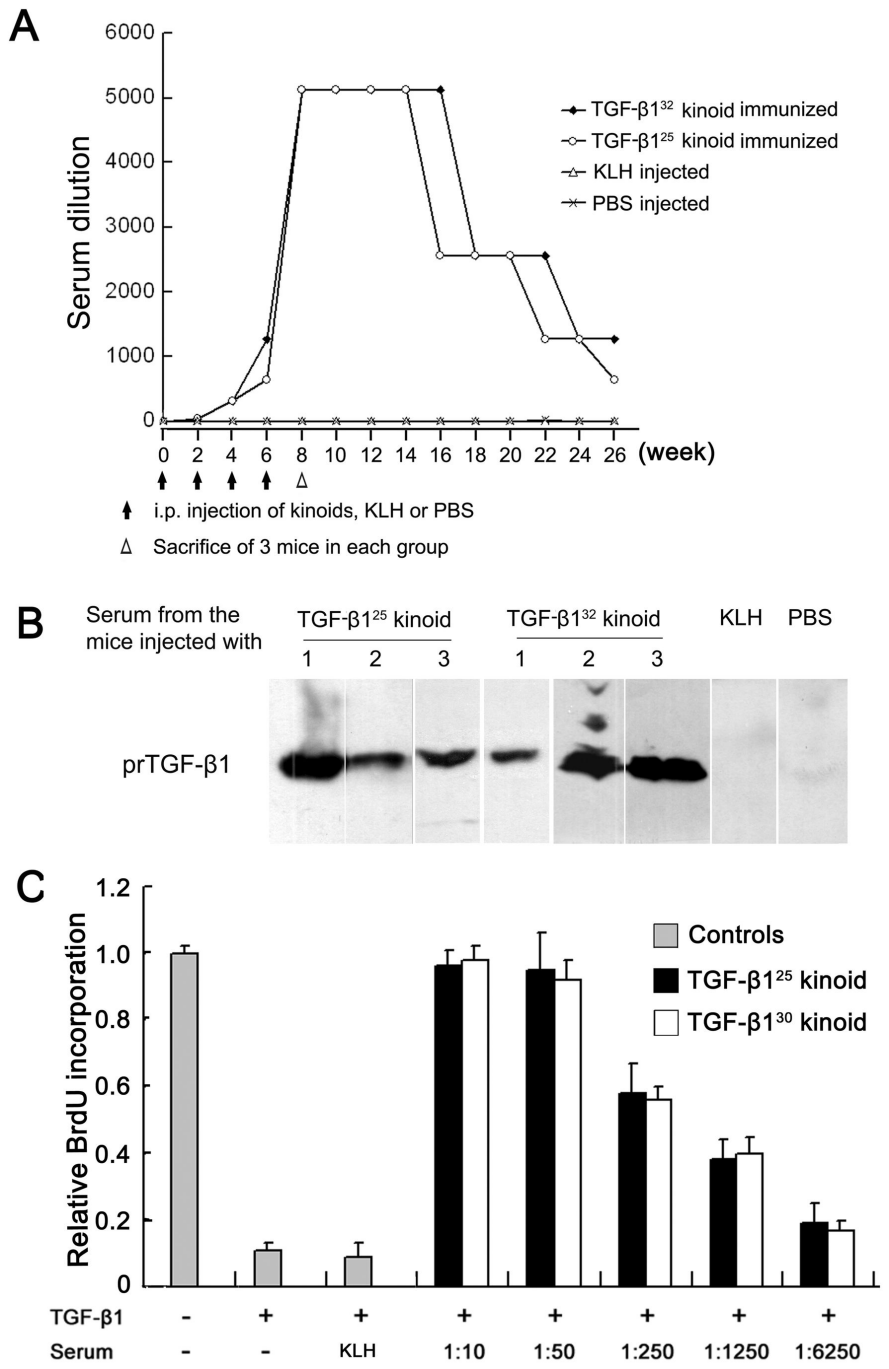
Immunization with TGF-β1 kinoids elicits TGF-β1 neutralizing IgG Abs. (A) Male BALB/c mice were i.p. injected with the TGF-β1 kinoids (50 μg in 0.2 mL), keyhole limpet hemocyanin (KLH, 50 μg in 0.2 mL) or an equal volume of phosphate-buffered saline, 2 weeks apart a total of four times. Indirect ELISA with recombinant human TGF-β1-coated (20 ng/well) plates showed that immunization with TGF-β1 kinoids stimulated high-titer and long-term anti–TGF-β1 IgG Abs. (B) Western blotting showed that four immunizations with TGF-β1 kinoids elicited the production of specific IgG Abs that could bind to membrane-bound recombinant human TGF-β1. A total of 5 μg of prokaryotically expressed human TGF-β1 (prTGF-β1) on each lane was applied to SDS-PAGE. The sera from the mice after 4 injections of TGF-β1 kinoids, KLH or PBS were used as the primary antibodies (1:800). (C) The antisera produced by TGF-β1 kinoid immunization showed dose-dependent neutralization activities on the TGF-β1-induced growth-inhibition on mink lung epithelial cells (Mv1Lu). After a 48-hour starvation in serum-free medium, Mv1Lu cells in 96-well plates were treated with TGF-β1 (2.5 ng/mL) and the various dilutions of the sera from the TGF-β1 kinoid-immunized or KLH-injected mice. Forty-eight hours post-treatment, BrdU incorporation in the cells was determined by quantitative ELISA. The incorporation of BrdU in the untreated Mv1Lu cells was set to 1. The results shown represent the mean ± the standard error of the mean (SEM) of three independent experiments, each of which was performed in triplicate wells. The error bars indicate SEM.

The results of direct ELISA showed that the first immunization only induced a low titer of antibodies. After the second injection, the Ab titers increased sharply. Four consecutive immunizations with the kinoids similarly elicited the production of large amounts of TGF-β1-specific Abs with the ELISA titers reaching 1:5120. These high titer Abs persisted for 4–6 weeks and then gradually declined. Six months after the first immunization, the anti–TGF-β1 Abs titers decreased to 1:640–1280 (Figure 1A). ELISA with TGF-β2- and TGF-β3-coated plates was also performed to evaluate the possible cross-reactions. The results indicated that the mouse serum after the fourth immunization only weakly cross-reacted with TGF-β2 and TGF-β3, with a positive reaction only being observed when the serum was diluted 1:20.

Western blotting was used to further verify the production of anti-TGF-β1 Abs. Western blotting revealed that a 1:800 dilution of the antiserum from the mice immunized with the TGF-β1 kinoids reacted with membrane-bound TGF-β1 (Figure 1B). These results clearly indicate that the two TGF-β1 kinoids possessed strong and similar TGF-β1-specific immunogenicities.

 The Mv1Lu cell growth assay showed that the mouse antiserum produced by the four injections of the kinoids had a similar, dose-dependent neutralization effect on the TGF-β1-induced growth-inhibition of Mv1Lu cells. The addition of 1:250 diluted mouse antiserum neutralized approximately 50% of the growth-inhibitory activity, whereas a 1:50 dilution almost completely abolished the growth-inhibitory activity of 2.5 ng/ml TGF-β1. In contrast, the sera from the KLH-immunized or PBS control mice had no effect (Figure 1C).

Additionally, after immunization, the mice showed no behavioral abnormalities. When the immunized mice were euthanized 6 months post-immunization, examination of the histological morphology of the vital organs of the immunized mice did not reveal any obvious adverse effects (data not shown).

### Vaccination against TGF-β1 protects mice from CCl_4_-induced hepatic fibrosis

**Figure 2:**
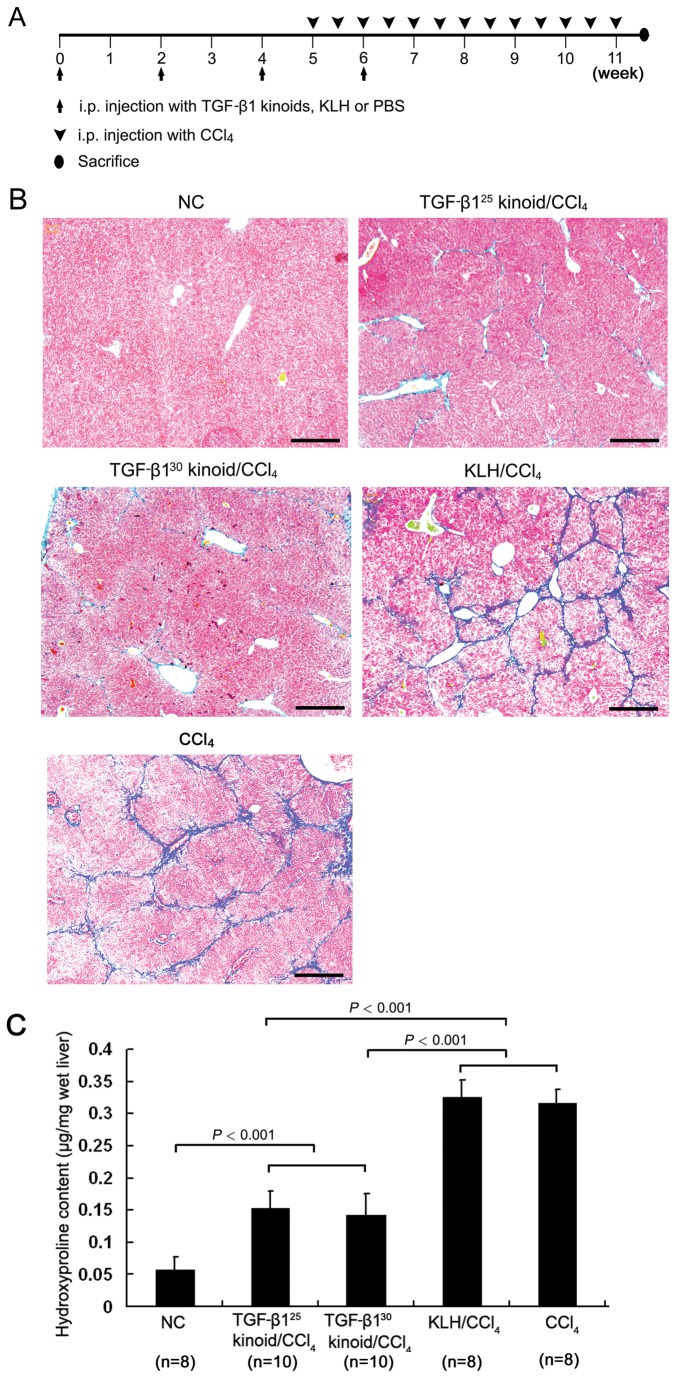
Vaccination with TGF-β1 kinoids suppresses CCl_4_-induced hepatic fibrosis in mice. The experimental protocol is illustrated in (A). After the immunization and subsequent 6-week i.p. CCl_4_ injection, the mouse liver tissues were evaluated for the deposition of fibrous tissues. Vaccination with TGF-β1 kinoids markedly attenuated CCl_4_-induced collagen deposition and architectural alteration (Masson’s trichrome staining, B) and reduced the hydroxyproline content (C) in the mouse liver. Bars = 200 μm. The error bars indicate SEM.

The TGF-β1 kinoid immunization and subsequent fibrosis induction were conducted following the protocol illustrated in Figure 2A. In this set of mice, the kinetic pattern of the anti–TGF-β1 Ab production was identical to that in the previous experiment, although the fourth boosting was performed one week after initiating the CCl_4_ injection. Masson’s trichrome staining of the liver sections showed that 6 weeks of repeated CCl_4_ injection resulted in obvious and uniform fibrous tissue deposition in the livers of the KLH/CCl_4_ and CCl_4_ mice. The severity of hepatic fibrosis in the two TGF-β1 kinoid vaccination groups was obviously milder than in the KLH/CCl_4_ and CCl_4_ groups (Figure 2B). Semi-quantitative evaluation by the Ishak system [34] followed by a statistical analysis indicated that the fibrosis scores of the two TGF-β1 kinoid vaccination groups were significantly lower than that of either the KLH/CCl_4_ or CCl_4_ groups, whereas there were no notable differences between the former two groups or the latter two groups (Table 2). 

**Table 2:** Grading of hepatic fibrosis in various groups.

Group	n	Histological score of hepatic fibrosis						
		0	1	2	3	4	5	6
NC	8	8	0	0	0	0	0	0
TGF-β1^25^ kinoid/CCl_4_*	10	0	2	5	2	1	0	0
TGF-β1^32^ kinoid/CCl_4_*	10	0	3	4	3	0	0	0
KLH/CCl_4_^#^	8	0	0	0	3	2	1	1
CCl_4_	8	0	0	0	4	1	2	1

*P < 0.05, vs. the KLH/CCl_4_ and CCl_4_ groups; ^#^ *P* > 0.05 vs. the CCl_4_ group. Mann-Whitney *U*-test.

As in the histological grading, the hepatic hydroxyproline content in the TGF-β1^25^ kinoid/CCl_4_ and TGF-β1^30^ kinoid/CCl_4_ groups were similar and significantly lower than that in either the KLH/CCl_4_ or CCl_4_ groups (Figure 2C). The data described above clearly indicate that vaccination against TGF-β1 with either the TGF-β1^25^ kinoid or the TGF-β1^30^ kinoid could significantly and similarly protect mice from CCl_4_-induced hepatic fibrosis.

Matrix metalloproteinases (MMPs), which are responsible for the degradation of ECM, are involved in hepatic fibrosis. In this study, we detected the expression of hepatic MMP-2 by using Western blot to explore the possible influence of the vaccination on the degradation of ECM in the fibrotic livers. The result showed that the expression of hepatic MMP-2 was up-regulated by chronic intoxication of CCl_4_ but the expression levels of MMP-2 had no significant difference among all the four CCl_4_ groups (Figure S1).

### Neutralization of TGF-β1 suppresses the activation of HSCs in vivo and in vitro

**Figure 3:**
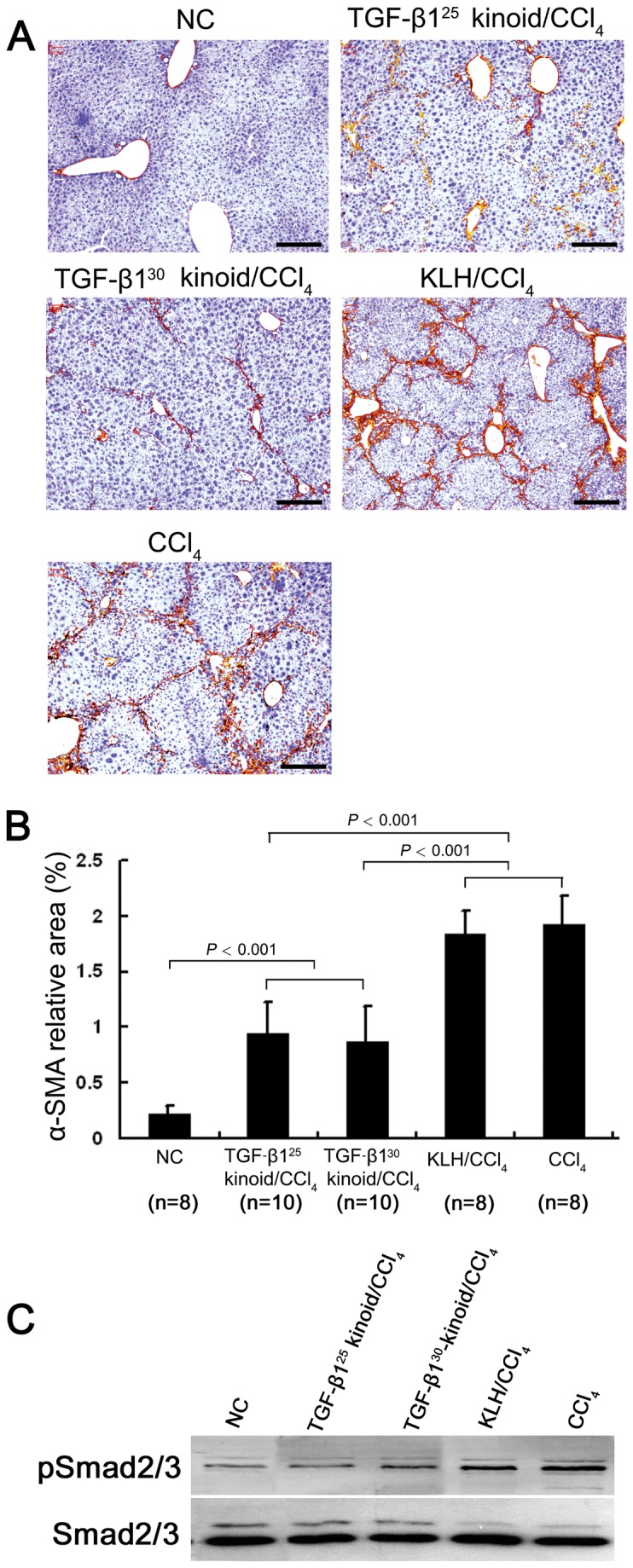
Vaccination with TGF-β1 kinoids suppresses HSC activation in CCl_4_-induced fibrotic mouse livers. The vaccination and CCl_4_ injection were performed as indicated in Figure 2A. After the 6-week CCl_4_ intoxication, immunohistochemistry for α-SMA (A) and subsequent quantitative computer-assisted morphometric analysis (B) demonstrated that the TGF-β1 kinoid vaccination significantly reduced the α-SMA-positive areas in the CCl_4_-induced fibrotic mouse livers. Bars = 200 μm. The error bars indicate SEM. (C) Western blotting showed that repeated CCl_4_ injection induced Smad2/3 phosphorylation (pSmad2/3), an indicator of TGF-β signaling activity, in the mouse liver tissues whereas vaccination with TGF-β1 kinoids attenuated Smad2/3 phosphorylation in the fibrotic liver tissues.

The expression of α-SMA, a typical marker of activated HSCs, and desmin, an indicator of intermediately differentiated HSC/MFB [36,37], was assessed by immunohistochemistry to evaluate the effect of vaccination with the TGF-β1 kinoids on HSC activation during hepatic fibrosis. Six weeks of CCl_4_ injections led to considerable increases in the amount of both α-SMA–positive cells distributed throughout the fibrotic septa and desmin-positive cells located at the rims of the fibrous septa (Figure 3A, Figure S2A). The computer-assisted semi-quantitative analysis revealed that both the TGF-β1^25^/CCl_4_ and TGF-β1^30^/CCl_4_ groups showed significantly decreased α-SMA- and desmin-positive areas compared with those in the CCl_4_ and KLH/CCl_4_ groups (*P* < 0.001), while there was no significant difference between either the former two groups or the latter two groups for both the α-SMA- and desmin-positive areas (Figure 3B, Figure S2B). Additionally, the detection of pSmad2/3, an indicator of TGF-β1 signaling, in the mouse liver tissues showed that the repeated CCl_4_ injections induced the phosphorylation of Smad2/3, whereas the vaccination with TGF-β1 kinoids attenuated the phosphorylation of Smad2/3 in the fibrotic livers (Figure 3C).

**Figure 4:**
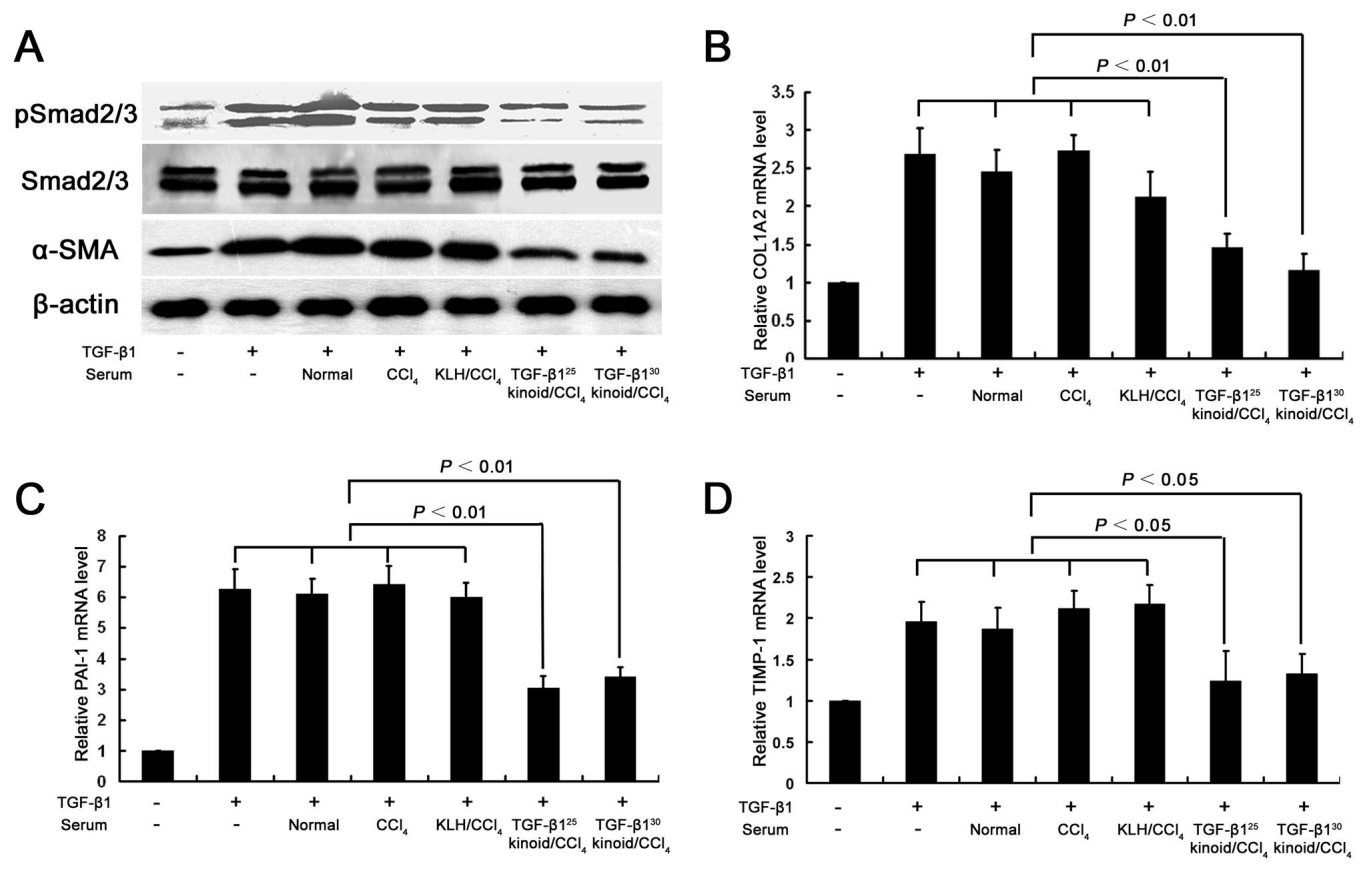
Antisera raised by TGF-β1 kinoid vaccination inhibit the TGF-β1-induced activation of pSmad2/3 and the expression of α-SMA, COL1A2, PAI-1 and TIMP-1 in HSC-T6 cells. HSC-T6 cells in 6-well plates were treated with TGF-β1 (6 ng/mL) and the mouse serum (1:200) for 24 hours. The phosphorylation of Smad2/3 and expression of α-SMA in were determined by Western blotting with the expression of Smad2/3 and β-actin serving as the internal controls (A). The expressions of COL1A2 (B), PAI-1 (C) and TIMP-1 (D) mRNA were evaluated by RT-qPCR. The RT-qPCR results represent the means ± the standard error of the mean of three independent experiments each of which was performed in triplicate qPCR reactions. The error bars indicate SEM.

 Our *in vitro* study with HSC-T6 reinforced the *in vivo* results. TGF-β1 at 6 ng/mL induced the increased phosphorylation of Smad2/3 and the upregulated expression of α-SMA, COL1A2, PAI-1 and TIMP-1. In contrast, antisera from the TGF-β1 kinoid-vaccinated mice at a 1:200 dilution significantly abolished these effects (Figure 4). These results indicate that the attenuation of CCl_4_-induced hepatic fibrosis is attributable to the suppression of HSC activation by TGF-β1 neutralization.

### Vaccination with TGF-β1 kinoids reduces apoptosis while accelerating the proliferation of hepatocytes in CCl_4_-induced fibrotic mouse liver

**Figure 5:**
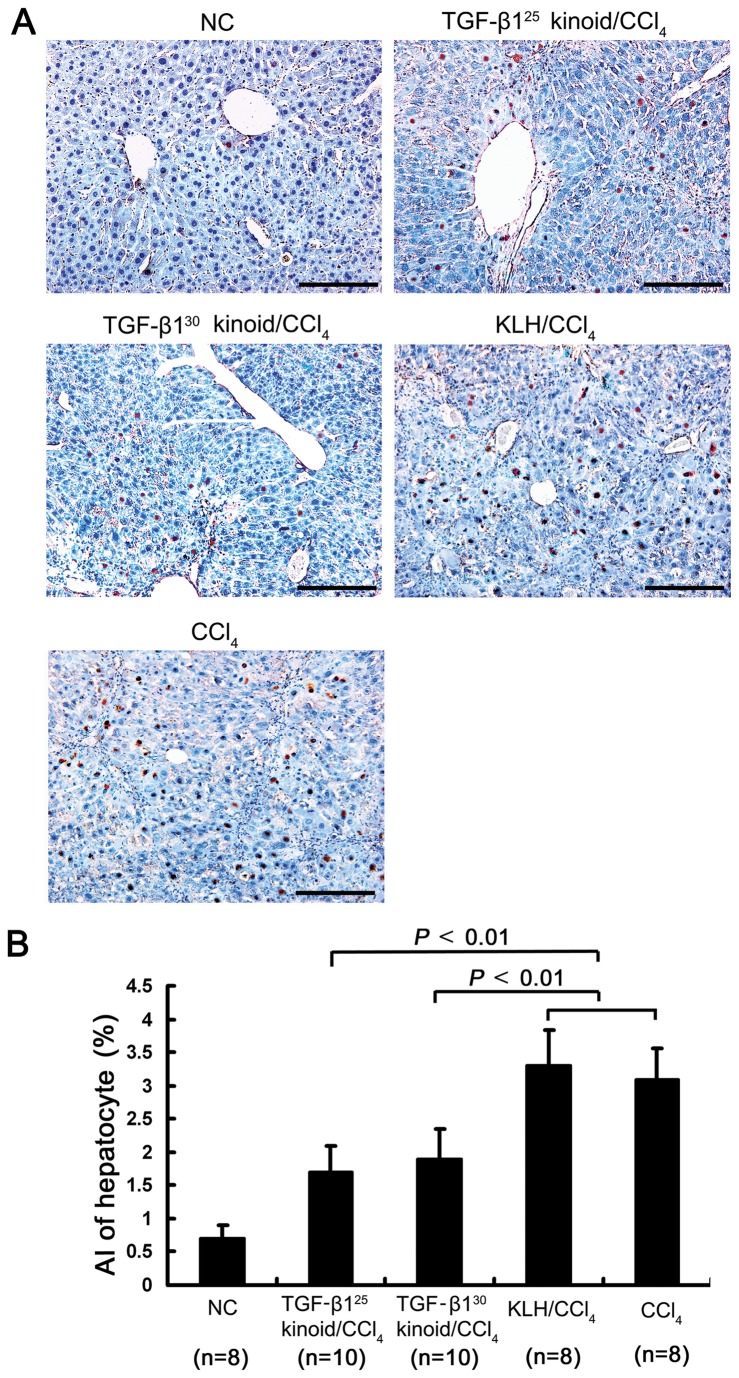
TGF-β1 vaccination reduces hepatocyte apoptosis in the fibrotic liver. After the vaccination and subsequent repeated CCl_4_ injections, the mouse livers were sectioned and the apoptotic cells were labeled using the TUNEL assay (A). The apoptosis index (AI) was evaluated and expressed as a percentage of the TUNEL-labeled hepatocytes out of the total number of hepatocytes counted. The repeated injections of CCl_4_ induced hepatocyte apoptosis, whereas vaccination with the TGF-β1 kinoids prior to CCl_4_ intoxication resulted in an alleviation of hepatocyte apoptosis in the fibrotic mouse livers (B). Scale bars = 100 μm. The error bars indicate SEM.

TUNEL staining showed that repeated injections of CCl_4_ drastically increased the percentage of apoptotic hepatocyte. The apoptotic cells were predominantly located around the fibrotic septa. Vaccination against TGF-β1 significantly reduced the proportions of apoptotic hepatocytes in the CCl_4_-induced fibrotic livers (Figure 5). These results suggest that the increased hepatocyte apoptosis in fibrotic livers is associated with TGF-β1 over-production and that neutralization of TGF-β1 protect hepatocytes from apoptosis.

**Figure 6:**
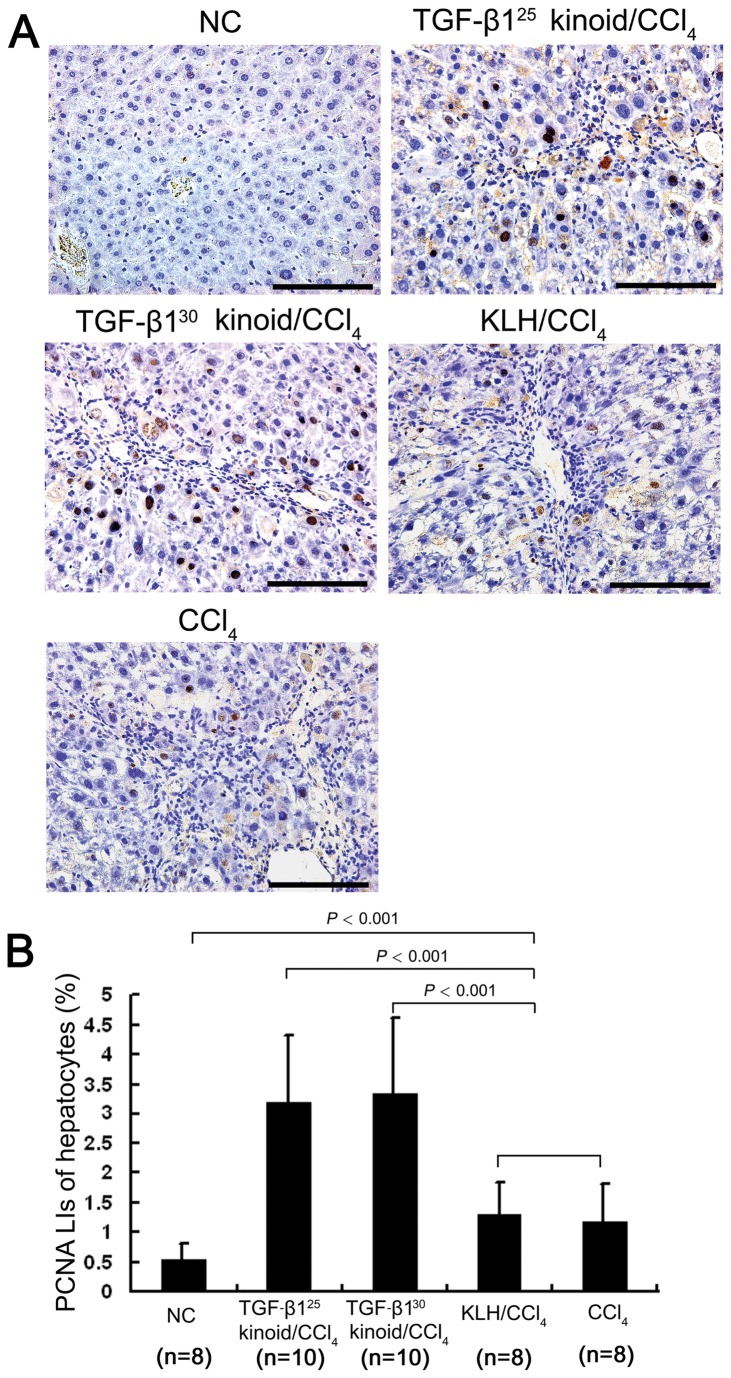
TGF-β1 vaccination accelerates hepatocyte proliferation in the fibrotic liver. Cell proliferation was determined by PCNA immunostaining (A). PCNA-positive nuclei were counted and expressed as a percentage of the total number of hepatocyte nuclei (PCNA labeling index, PCNA LI) (B). Repeated CCl_4_ injections induced elevated hepatocyte proliferation with PCNA-positive hepatocytes predominantly distributed around the fibrous septa, whereas vaccination with the TGF-β1 kinoids led to further increases in the percentages of the PCNA-expressing hepatocytes in mouse livers. Scale bars = 100 μm. The error bars indicate SEM.

The repeated CCl_4_ injections led to a marked increase in the number of PCNA-positive hepatocytes in all four of the CCl_4_-treated groups, with a distribution pattern similar to that of PCNA-immunostaining (Figure 6). Moreover, among these four groups, the two TGF-β1 kinoid vaccination groups showed further significant increases in the percentages of PCNA-positive hepatocytes compared with either the CCl_4_ or the KLH/CCl_4_ group (Figure 6A, B), indicating that neutralization of the excessive TGF-β1 through inoculation with the TGF-β1 kinoids accelerated the proliferation of hepatocytes in the fibrotic livers.

## Discussion

It has been verified that TGF-β1 and platelet-derived growth factor B (PDGF-B) are the two most important pro-fibrogenic cytokines in hepatic fibrosis. In a previous study, we tested the preventive effect of vaccination against PDGF-B on experimental fibrosis. The results showed that immunization with PDGF-B kinoids significantly prevented the development of CCl_4_-induced hepatic fibrosis in mice [38]. In the current study, we demonstrated that active vaccination against TGF-β1 with two TGF-β1 kinoids significantly attenuated CCl_4_-induced hepatic fibrosis in BALB/c mice. To our knowledge, this study is the first attempting to suppress hepatic fibrosis by abolishing TGF-β1 through an active immunization measure. 

B lymphocytes that recognize self-proteins have been shown to be retained and continue to produce trace levels of autoantibodies that can participate in immune regulation, even after the maturation of the immune system. The immune tolerance to self-proteins is due to the prohibition or deletion of the corresponding T helper cells that are responsible for providing a second co-stimulatory signal. The B cells can be activated to produce autoantibodies, provided the T helper cell signal is present along with an autoantigen. To break the immune tolerance to self-proteins, such as pathogenetically relevant cytokines, the self-proteins can be linked to T helper epitope-rich carrier proteins [31]. Cross-linking the self-proteins or their epitopes to carrier proteins, covalently or non-covalently, has been verified to be a simple, time-saving, reliable strategy for the preparation of autoimmunogens [24,26]. Moreover, the epitope-carrier heterocomplexes generally have high immunogenicities because high-density polypeptides are bound to the carrier proteins. In the current study, we prepared two TGF-β1 kinoids by cross-linking two TGF-β1 epitopes, TGF-β1^25^–(41–65) and TGF-β1^30^–(83–112), which have been reported to be the key domains mediating TGF-β1-receptor interactions [32,33], with the carrier protein KLH and then immunized mice with these kinoids. Our results demonstrated that four injections with these kinoids elicited high titers of anti-TGF-β1 Abs that have quite weak cross-reactivities with the other two TGF-β family members with high homology to TGF-β1, TGF-β2 and TGF-β3. Furthermore, the antisera could both reverse the TGF-β1-induced growth-inhibition on Mv1Lu cells. These data indicate that the two TGF-β1 kinoids could elicit the production of TGF-β1 neutralizing Abs and could serve as TGF-β1 vaccines for further experiments.

In this study, analysis of the phosphorylation of Smad2/3, an indicator of the TGF-β1 signaling, showed that the TGF-β1 kinoids vaccination suppressed the phosphorylation of Smad2/3 in the fibrotic mouse liver tissues. Moreover, the *in vitro* study showed that the post-immunization mouse serum inhibited the TGF-β1-induced Smad2/3 phosphorylation, α-SMA, COL1A2, PAI-1 and TIMP-1 expression in HSC-T6 cells. These results clearly indicate that the suppression of CCl_4_-induced hepatic fibrosis in the vaccinated mice is attributable to the blockage of TGF-β1 signaling and further attenuation of the activation of HSCs. These results are within our expectation, given that several experiments have demonstrated the inhibitory effects of anti–TGF-β1 Abs (passive immunization) on fibrosis [8,9,39]. Although the mechanisms for suppressing hepatic fibrosis by vaccination against TGF-β1 and by the direct injection of anti–TGF-β1 Abs are similar, the advantages of active immunization over Ab injection are obvious: the preparation of vaccines are simpler and less expensive than specific Abs, the application of vaccines are convenient, and the production of anti–TGF-β1 Abs after vaccination is constant and long-lasting, avoiding the fluctuation of the Ab concentrations in the circulation when intermittently injected. Therefore, vaccination against TGF-β1 is theoretically more feasible than the utilization of anti–TGF-β1 Abs and, possibly, the other presently available measures.

An important finding in this study is that the prevention of fibrosis through vaccination against TGF-β1 was accompanied by a decrease in hepatocyte apoptosis and an increase in hepatocyte proliferation. The interpretations of these results might be as follows. On the one hand, TGF-β1 has pro-apoptotic and anti-proliferative effects on epithelial cells such as hepatocytes [4,5]; thus, the neutralization of excessive amounts of TGF-β1 can attenuate the apoptosis and promote the regeneration of injured hepatocytes. On the other hand, the attenuation of fibrosis improves the exchange of molecules between the sinusoidal space and the hepatocytes, thereby inhibiting the apoptosis and facilitating the regeneration of hepatocytes. The chronic loss of functional hepatocytes, along with fibrogenesis, is an important pathological alteration in chronic liver diseases. Attenuating the apoptosis and promoting the regeneration of hepatocytes by TGF-β1 vaccination will help restore the structure and function of the liver.

Although in this study we immunized the mice before initiating the CCl_4_ injections, which is “preventive” rather than “therapeutic” for experimental hepatic fibrosis, we believe this measure will also be applicable for the treatment of hepatic fibrosis. One of the main concerns regarding the use of therapeutic vaccines for hepatic fibrosis is whether the vaccines are able to elicit Ab production in animals and later on, in patients, with pre-existing hepatic fibrosis. The humoral immune response in animals and in patients with hepatic fibrosis is not seriously impaired, leaving them capable of producing Abs in response to immunization [40]. This question has been partially settled in our present study, which showed that boosting immunization with TGF-β1 kinoids led to a further increase in the Ab levels after the initiation of CCl_4_ injections. However, we suggest that the therapeutic vaccination should be delivered as early as possible. 

One of the major concerns about the safety of vaccination against cytokines is that the neutralization of the cytokines by autoantibodies might impair non-targeted healthy tissues because cytokines are highly pleiotropic. This adverse effect was not observed in our current study or in investigations by others [19-30]. Nevertheless, the safety of anti–TGF-β1 vaccination, especially given the concerns of its potential influences on the immune ability and carcinogenesis, still needs to be investigated carefully in future studies. 

The results of this study also provide some inspirations. Because some other cytokines, such as PDGF and CTGF, are also involved in fibrotic disorders and fibrotic disorders of various organs and tissues share a rather common mechanism, the combined vaccination against more than one of these pro-fibrotic cytokines might result in a more effective inhibition of fibrosis and should be considered for fibrotic disorders.

In conclusion, our current study provides a novel, simple and potentially feasible approach for inhibiting hepatic fibrosis via active vaccination against TGF-β1. Although further investigations are required, this strategy might be developed into a clinically feasible therapeutic approach for the treatment of hepatic fibrosis.

## Supporting Information

Figure S1 **The vaccination did not significantly influence the expression of MMP-2 in CCl_4_-induced fibrotic mouse livers.** (A) A representative image of Western blot detection of hepatic MMP-2 expression in various groups. (B) Semi-quantitatively analysis of the expression of MMP-2 in the mouse livers.
(TIF)

Figure S2 **Vaccination with TGF-β1 kinoids suppresses HSC activation in CCl_4_-intoxicated fibrotic mouse livers as indicated by desmin immunostaining.** BALB/c mice were immunized with TGF-β1 kinoids or injected with KLH or PBS, followed by i.p. injection of CCl_4_ (1 mL/kg) twice a week for 6 weeks. Then the mouse livers were fixed and immunohistochemically stained for desmin (A). Quantitative computer-assisted morphometric analysis of the desmin immunostaining (B) demonstrated that TGF-β1 kinoids vaccination significantly reduced the desmin-positive areas in CCl_4_-induced fibrotic mouse livers. Bars = 200 μm. Error bars indicate SEM.
(TIF)
