# Supplementary figures and images for: Attenuation of CCl4-Induced Hepatic Fibrosis in Mice by Vaccinating against TGF-β1
Source: PLoS One. 2013 Dec 11;8(12):e82190. doi: 10.1371/journal.pone.0082190 (PMC3859579; doi:10.1371/journal.pone.0082190)

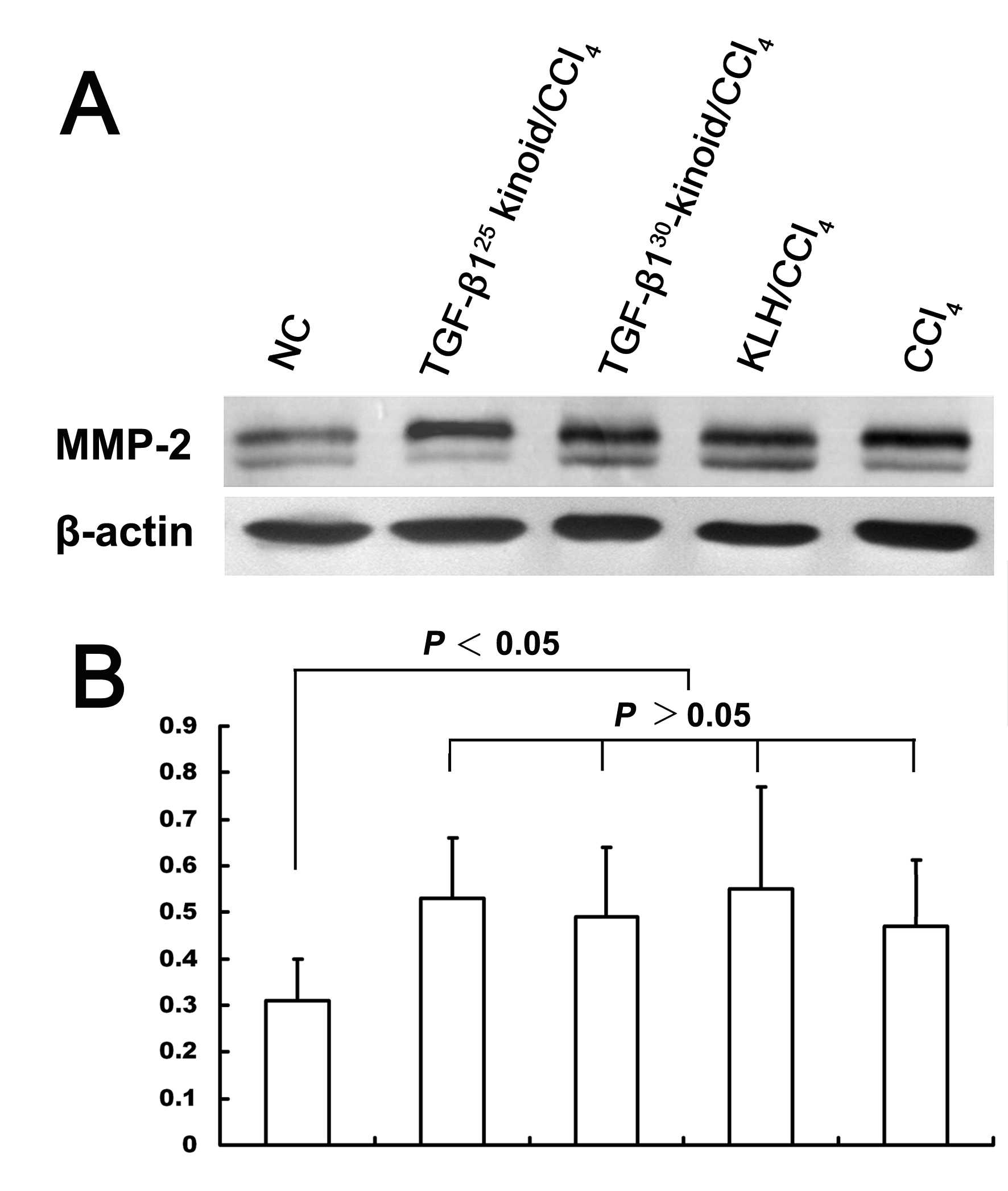

Supplement: Figure S1 — The vaccination did not significantly influence the expression of MMP-2 in CCl4-induced fibrotic mouse livers. (A) A representative image of Western blot detection of hepatic MMP-2 expression in various groups. (B) Semi-quantitatively analysis of the expression of MMP-2 in the mouse livers. (TIF) [file pone.0082190.s001.tif]

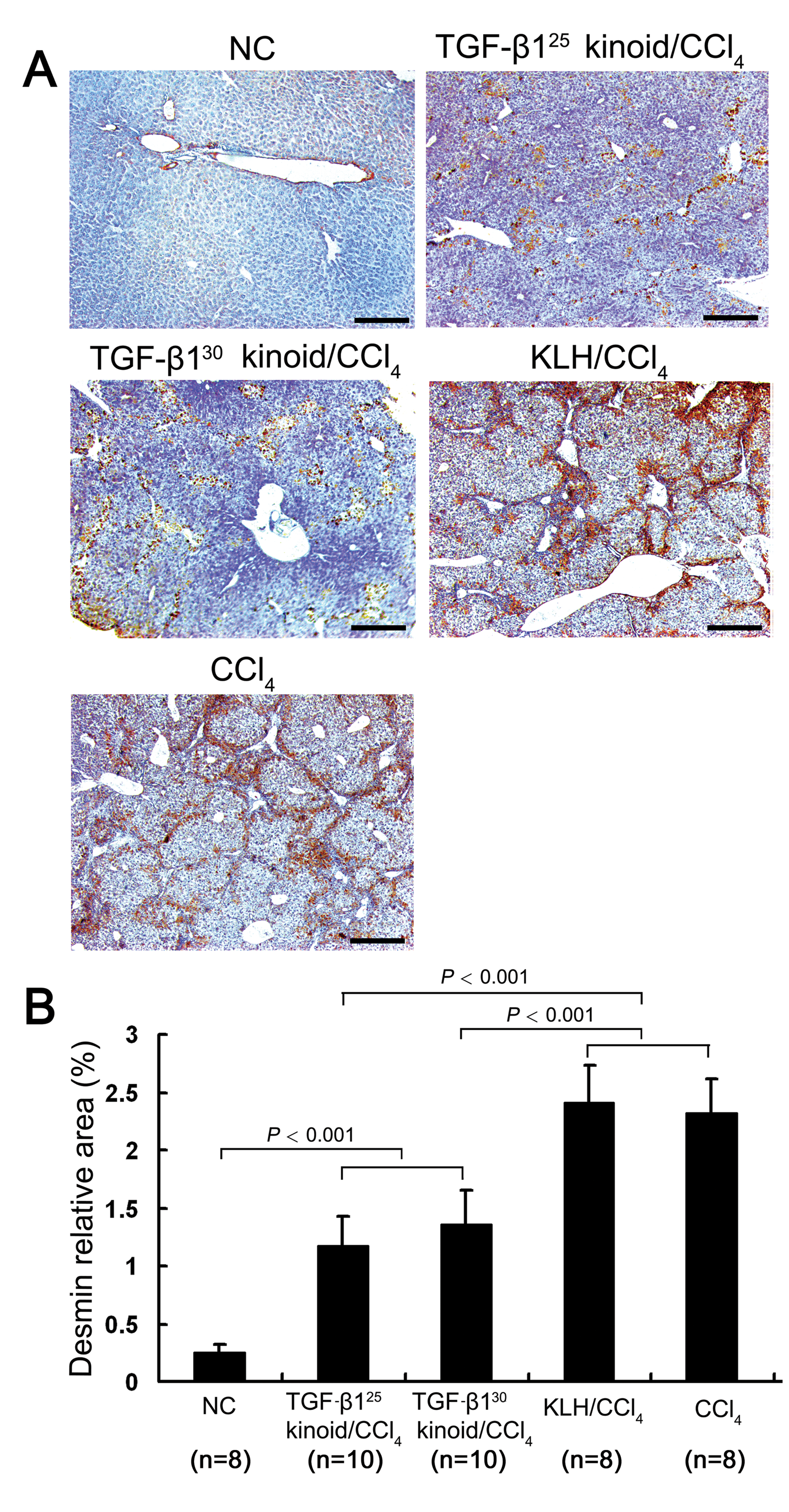

Supplement: Figure S2 — Vaccination with TGF-β1 kinoids suppresses HSC activation in CCl4-intoxicated fibrotic mouse livers as indicated by desmin immunostaining. BALB/c mice were immunized with TGF-β1 kinoids or injected with KLH or PBS, followed by i.p. injection of CCl4 (1 mL/kg) twice a week for 6 weeks. Then the mouse livers were fixed and immunohistochemically stained for desmin (A). Quantitative computer-assisted morphometric analysis of the desmin immunostaining (B) demonstrated that TGF-β1 kinoids vaccination significantly reduced the desmin-positive areas in CCl4-induced fibrotic mouse livers. Bars = 200 μm. Error bars indicate SEM. (TIF) [file pone.0082190.s002.tif]
